# Recent inner ear specialization for high-speed hunting in cheetahs

**DOI:** 10.1038/s41598-018-20198-3

**Published:** 2018-02-02

**Authors:** Camille Grohé, Beatrice Lee, John J. Flynn

**Affiliations:** 1 Division of Paleontology, American Museum of Natural History, Central Park West at 79th Street, New York, NY 10024 USA; 2 Middlebury College, Middlebury, VT 05405 USA; 3 Richard Gilder Graduate School, American Museum of Natural History, Central Park West at 79th Street, New York, NY 10024 USA

## Abstract

The cheetah, *Acinonyx jubatus*, is the fastest living land mammal. Because of its specialized hunting strategy, this species evolved a series of specialized morphological and functional body features to increase its exceptional predatory performance during high-speed hunting. Using high-resolution X-ray computed micro-tomography (μCT), we provide the first analyses of the size and shape of the vestibular system of the inner ear in cats, an organ essential for maintaining body balance and adapting head posture and gaze direction during movement in most vertebrates. We demonstrate that the vestibular system of modern cheetahs is extremely different in shape and proportions relative to other cats analysed (12 modern and two fossil felid species), including a closely-related fossil cheetah species. These distinctive attributes (i.e., one of the greatest volumes of the vestibular system, dorsal extension of the anterior and posterior semicircular canals) correlate with a greater afferent sensitivity of the inner ear to head motions, facilitating postural and visual stability during high-speed prey pursuit and capture. These features are not present in the fossil cheetah *A. pardinensis*, that went extinct about 126,000 years ago, demonstrating that the unique and highly specialized inner ear of the sole living species of cheetah likely evolved extremely recently, possibly later than the middle Pleistocene.

## Introduction

The cheetah *Acinonyx jubatus* is a large, anatomically-specialized, cursorial felid carnivoran, and the fastest living land mammal. Over short distances, cheetahs can reach a maximum speed of 25.9–29 m/s^1,2^. The species usually hunts swift ungulates such as gazelles, antelopes, and impalas by chasing them at high speeds, knocking them over, and biting their throats until they suffocate^3,4^. Given its lightly-built body compared to other felids, the cheetah captures medium-sized prey, weighing only 23 to 56 kg on average, which limits the risk of injuries and provides enough time for the animal to eat before the arrival of kleptoparasites such as lions, leopards, hyaenas or vultures^4,5^. Because of this distinctive hunting strategy, the species evolved an array of morphological characteristics related to fast motion and enhanced predatory performance^3,6–9^. The genome sequence of cheetahs also revealed positive selection of genes mediating energy metabolism and regulating muscle contraction, involved in their high-speed adaptations^10^.

No prior study has investigated the sensory capacities of cheetahs through analyses of the anatomy of their intracranial organs, including the inner ear, which facilitates their remarkable capacity of maintaining visual and postural stability while running and capturing prey at high speeds, a key feature in the hunting specialization of the modern cheetah species. The inner ear, located in the petrosal bone at the base of the occipital region, plays a primary role in detecting head movements (accelerations and decelerations in both linear and rotational directions – pitch, yaw, and roll) and directly guiding the eyes and neck muscles in order to maintain posture and stabilize balance and gaze (e.g., vestibulo-ocular reflex^11,12^). The bony labyrinth surrounds the soft tissues of the inner ear and can be used as a proxy for investigating the shape and dimensions of this organ. The morphology of the bony labyrinth can be correlated with phylogenetic relationships in some groups^13–18^. The vestibular system within the bony labyrinth, in particular, also can reflect ecological capabilities of vertebrates, especially their locomotor styles^19–26^. In this paper, we investigate the shape and the dimensions of the bony vestibular system in a number of individual extant cheetahs (taking into account variation that might be present in members of several subspecies) compared to other extant felids, using high-resolution μ-CT data and 3D morphometric analyses. Because of the long evolutionary history of the cheetah lineage^27^, and to provide greater insights into the timing of its specialized predatory behavior, we studied the fossil ‘giant’ cheetah *Acinonyx pardinensis* (early-middle Pleistocene) and one of the earliest felids *Proailurus lemanensis* (Oligo-Miocene of Eurasia^28^), to assess the ancestral inner ear condition for the cheetah and felid clades (Supplementary Fig. 1; Supplementary Tables 1–2; see Methods).

We predict that the extant cheetah species will display a greater volume of its vestibular system, when compared to other felids, given the primary role of the vestibular system in sensing head motions and enabling co-ordination of body movement and gaze direction, essential features for high-speed running (e.g., enlarged utricular volume increasing semicircular canal system afferent sensitivity^29^). We investigated the variation of the bony vestibular system shape among felids using 3D geometric morphometrics, a powerful method for determining phylogenetic and functional patterns of mammalian inner ears^14,18,26,30–33^. Among the shape factors previously identified as related to the sensitivity of the vestibular system afferents are the size of the semicircular canals, and the variations of orthogonality between canals and their degree of out-of-plane curvatures^21,26,34–38^. We would expect that fossil and extant cheetahs will share similarities in shape and dimensions due to their close shared ancestry and potentially similar locomotor and predatory ecologies indicated by prior skeletal studies^39–42^. In contrast, we would expect different vestibular system shapes and dimensions between extinct and modern cheetahs, if they adopted different hunting strategies or degrees of high-speed locomotor performance. These differences would indicate a lag in modification of the inner ear relative to the locomotor system in the evolution of high-speed hunting in this lineage.

## Results

### Volume of the vestibular system

**Figure 1:**
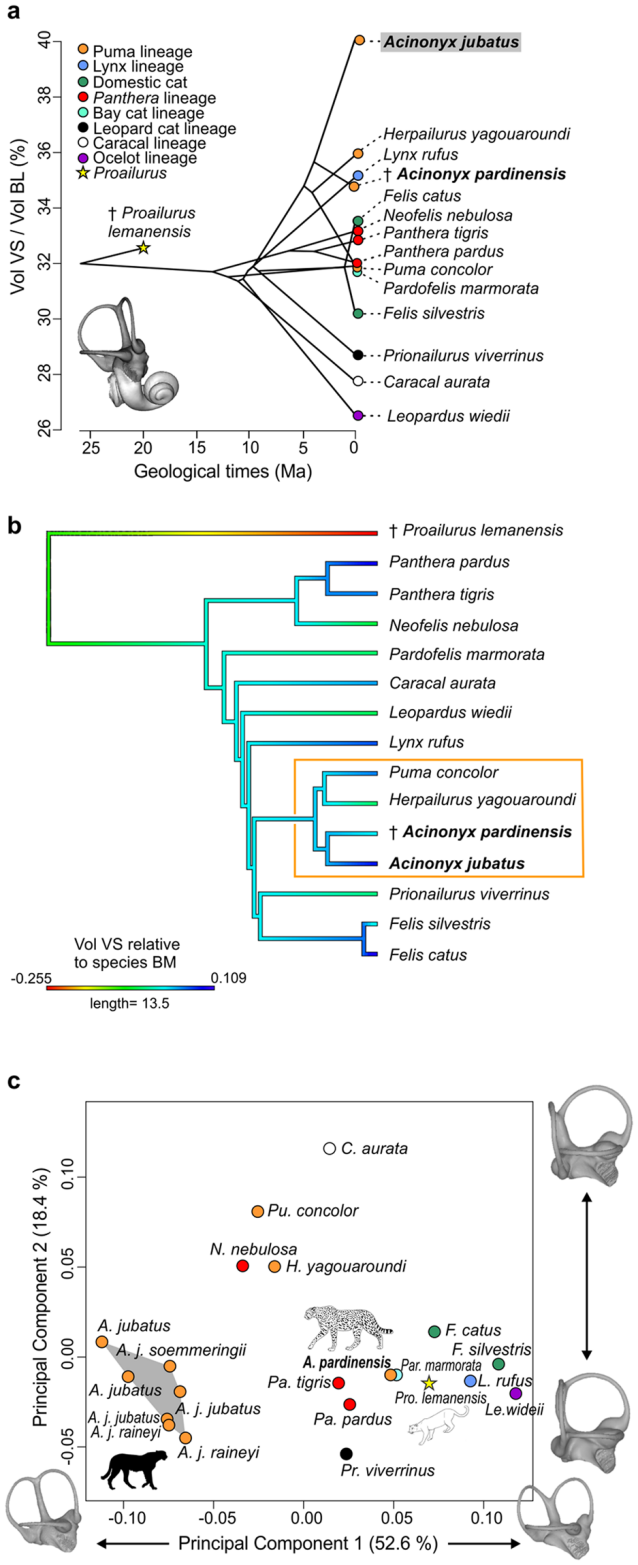
Vestibular System (VS) proportions and shape of seven extant cheetah specimens compared to 14 other extant and extinct felids. (**a**) Representation in phenogram of the Volume of the Vestibular System (Vol VS, illustrated in dark grey) relative to the entire Bony Labyrinth Volume (Vol BL) using phylogeny of felids (Supplementary Fig. 1 and Supplementary Table 1). (**b**) Residuals of the regression model between Vol VS and estimated body mass of felid species, represented as a color gradient along phylogeny. (**c**) Principal Components Analysis (PCA) of 3D shape. Note that VS of extant cheetahs has a highly distinct shape on PC1 (associated, among others, with longer anterior and posterior semicircular canals), its relative volume is much larger compared to any other felids, and also larger than expected from its estimated body mass, especially when compared to other puma-lineage felids and its nearest relative, the fossil cheetah *Acinonyx pardinensis*. Also note the primitive shape and proportions of VS in the fossil cheetah *A. pardinensis*, resembling that of the early fossil felid *Proailurus lemanensis*.

The volume of the vestibular system relative to the volume of the entire bony labyrinth in felids varies between 26.5 and 39.9% (Fig. 1a, Supplementary Table 3; see Methods). The mean volume of 32.4% for all extant felids is very close to that of the earliest fossil felid *Proailurus*, thus representing a primitive condition for the clade. The *Panthera* lineage, the earliest-diverging lineage of extant felids (represented in our sample by the tiger, leopard, and clouded leopard), is closely clustered with the extinct *Proailurus*, and the extant puma and marbled cat. The domestic cat (*Felis catus*) and its putative ancestor, the wild cat (*Felis silvestris*), differ markedly, with the domestic cat having the larger vestibular system of the two species. The smallest volume percentages occur in the margay, golden cat, and fishing cat (26.5, 27.7, and 28.7%, respectively), whereas, for taxa with higher than average volumes, vestibular systems are progressively larger in the fossil cheetah, bobcat, jaguarondi, and at the far extreme, the extant cheetah. The average volume of the vestibular system in *A. jubatus* represents a remarkable 39.9% of the bony labyrinth volume, or 1.5 times greater than the percentage volume for the margay. This percentage ratio varies between 38 and 43.7% for the 7 extant cheetah specimens studied, each individual of which has a relatively larger vestibular system than any other felid in our sample (Supplementary Table 3). Within the puma lineage, the vestibular system ratio of *Puma concolor* is nearly 4% lower compared to its closest relative *Herpailurus yagouaroundi*, and the ratio for this system is between 2 and 8% lower in the fossil cheetah compared to modern cheetah individuals. The fossil cheetah vestibular volume also is smaller than in the jaguarondi, but larger than in the puma (Supplementary Table 3). Relative to estimated body masses of each specimen analysed, using another proxy for body mass (basicranial length), the volume of the vestibular system of *Proailurus* is much smaller than predicted from the overall felid regression model (Fig. 1b, Supplementary Table 4; see Methods). From this ancestral condition, extant felids evolved larger vestibular systems relative to their body masses. Within the puma clade, the extant cheetah shows the greatest increase of the vestibular system volume relative to its body mass, and it is far larger than in its closest relative, the fossil ‘giant’ cheetah *Acinonyx pardinensis*.

### 3D shape of the vestibular system

We performed a Principal Components Analysis (PCA) based on Procrustes coordinates generated from a three-dimensional landmark dataset placed on the vestibular systems of our felid sample (Supplementary Table 5; see Methods). The first two axes of the PCA explain nearly 75% of the shape variation of the vestibular system (Fig. 1c; PC1: 52.6%, PC2: 18.4%). PC1 separates the shape of the vestibular system of extant cheetahs and, to a lesser extent, that of the clouded leopard, puma, and jaguarondi, with negative values, from the rest of the studied felids (including the two fossils) that have positive values. The margay exhibits the most distinct shape on PC1 relative to extant cheetahs. Negative PC1 values are associated with: longer and straighter common crus, and therefore anterior and posterior semicircular canals (SC) more extended dorsally (Supplementary Fig. 2a,c,d); increase of the angle between the anterior and posterior SC (Supplementary Fig. 2b); anterior portion of the anterior SC twisted laterally (Supplementary Fig. 2b**)**; lateral portion of the posterior SC twisted posteriorly (Supplementary Fig. 2b); straighter and narrower lateral SC (Supplementary Fig. 2a,b,d**)**; more medial position of the bifurcation between the lateral and posterior SC (Supplementary Fig. 2b); and greater ventral extension of the posterior SC (Supplementary Fig. 2c). Positive PC1 values are associated with: shorter common crus; anterior and posterior SC widths larger than heights; decreased angle between the anterior and posterior SC, with less twisted anterior and posterior SC; wider lateral SC, concave in lateral view; more lateral bifurcation between the lateral and posterior SC; and less ventral extension of the posterior SC (Supplementary Fig. 2a–d).

PC2 further distinguishes the shape of the vestibular system of the golden cat and, to a lesser extent, of the puma, jaguarondi, and clouded leopard, with positive values, from those of most other felids, particularly the fishing cat, with negative values (Fig. 1c). Also, the shape of the vestibular system in the subspecies of extant cheetahs are mostly distinguished on PC2, with the Tanzanian and South African forms exhibiting more negative scores compared to the Northeast cheetah. Negative PC2 values are associated with: longer and slightly curved common crus; anterior SC slightly more extended dorsally; narrower anterior and posterior SC; straighter lateral SC; and posterior SC less extended ventrally (Supplementary Fig. 3). Positive PC2 values are associated with: shorter and straighter common crus; slightly less dorsally extended anterior SC; wider anterior and posterior SC; more curved lateral SC; posterior SC extending more ventrally (Supplementary Fig. 3).

**Figure 2:**
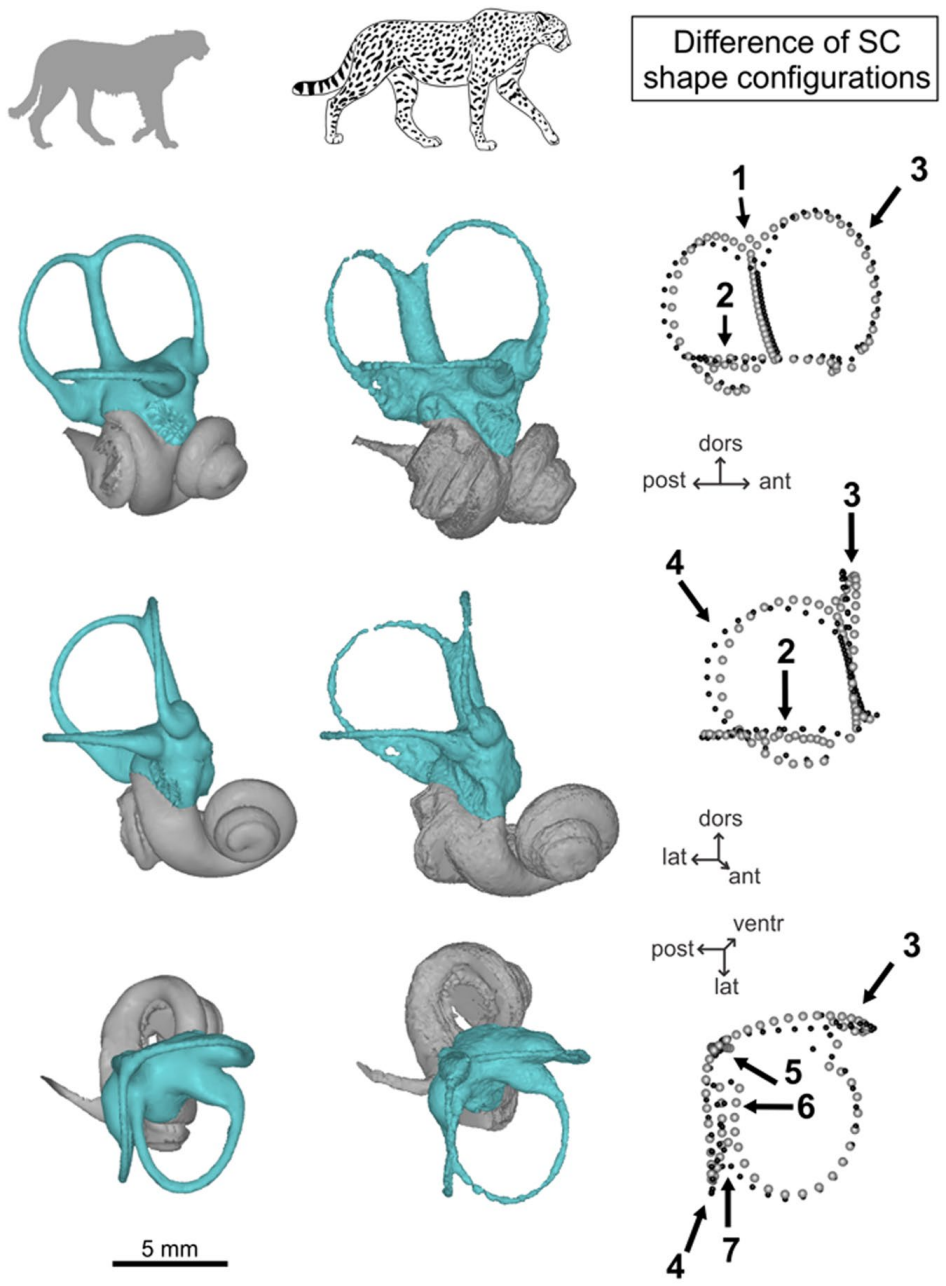
Shape differences of the Vestibular System (VS, highlighted in blue) between the fossil and extant cheetah species. VS in lateral, anterolateral, and dorsal views from top to bottom. Black points = extinct *Acinonyx pardinensis* Procrustes coordinates; grey points = extant *Acinonyx jubatus* mean Procrustes coordinates. Numbers correspond to main regions of shape differences: 1, length of the common crus; 2, out-of-plane curvature of the lateral semicircular canal (SC); 3, shape of the anterior SC; 4, shape of the posterior SC; 5, angle between the anterior and posterior SC; 6, width of the lateral SC; 7, position of the bifurcation between the lateral and posterior SC.

The fossil cheetah displays a distinct shape from the extant cheetahs on PC1 (Fig. 1c). Relative to the Procrustes coordinates of the Pleistocene fossil cheetah, the mean Procrustes coordinates for modern cheetahs indicate differences in the length of the common crus (longer), shape of the semicircular canals (anterior SC less extended anteriorly, posterior SC less extended posteriorly, both anterior and posterior SC more extended dorsally, narrower lateral SC), out-of-plane curvature of the lateral SC (straighter), angle between the anterior and posterior SC (greater), and position of the bifurcation between the lateral and posterior SC (more medial, resulting in a greater separation between the posterior branch of the lateral SC and the ventral branch of the posterior SC) (Fig. 2).

To test the allometric effect of body size on vestibular system shape variation, we performed a Procrustes ANOVA between log-transformed centroid size and Procrustes coordinates of the vestibular system of felid species (see Methods). Associated permutation tests indicate no statistically significant covariation between body size and vestibular shape data (p-value = 0.098). Moreover, we tested the influence of phylogeny or evolutionary relatedness of felid species on our sample of vestibular system shapes using the K*mult* statistic (see Methods) and we found a significant, but rather weak, phylogenetic signal in our shape data (p-value = 0.02, K*mult* = 0.496).

## Discussion

The documentation of a much greater volume of the vestibular system relative to the overall bony labyrinth volume in the extant cheetah among all 15 species sampled across the felid clade, as well as one of the largest vestibular systems relative to estimated body mass in felids, including the largest vestibular system within the puma clade and much larger than its nearest relative the Pleistocene fossil cheetah, constitute previously unknown findings related to the extraordinarily fast motion during prey pursuit in the modern cheetah. This suggests an enhanced role of the two vestibular sensory components, otolith organs and semicircular canals, during the evolutionary history of cheetahs (e.g., enlarged utricular volume increasing the semicircular canal system afferent sensitivity^29^). The otolith organs (saccule and utricule) ensure the detection of linear accelerations and decelerations of the body, as well as gravity changes, essential in cheetahs to maintain balance while accelerating in forward and lateral directions during high-speed pursuit and rapid decelerations associated with prey capture (as associated with hunting dynamics in wild cheetahs^2^). Both the otolith organs and the semicircular canals, which sense rotational head movements (pitch, roll, yaw), play a critical role for maintaining gaze direction and head posture while the animal is maneuvering and changing directions during high-speed runs.

Our 3D shape analysis of the vestibular system revealed that phylogenetic relationships among felids significantly influence its shape variation, whereas a key ecological attribute, body size, does not. However, the low K*mult* value for the significant phylogenetic correlation suggests that other factors (i.e., physiological and functional) also control the vestibular system shape in felids. The results document that the seven members of the extant cheetah subspecies all display more elongated anterior and posterior semicircular canals (ASC, PSC) than any other felid, due to the presence of a longer common crus, while the lateral semicircular canal (LSC) is narrower and less elongated. Longer canals are associated with greater afferent sensitivity to rotational head movements in mammals^21,29,36,43^. Previous studies suggested that both the ASC and PSC influence vestibulo-ocular and vestibulo-collic reflexes, which are responsible for adjustments of eye direction and head position during locomotion, while LSC dimensions primarly correlate with navigation control, particularly important for species that locomote in complex 3D environments, such as arboreal, aquatic, and aerial specialists^21,37,44^. Thus, the observed configuration of the SC in all specimens of the extant cheetah analysed indicate greater afferent sensitivity of the vestibular system to pitch and roll head movements, ensuring the activation of the visual and postural reflex adjustments during the low amplitude vertical head motion associated with high-speed hunts. These specialized hunters exhibit a greater angle between the ASC and PSC, deviating from an orthogonal position, and out-of-plane curvatures of the vertical SC, compared to the vestibular systems of other felids. The deviation from orthogonality of the SC has been associated with slower overall head rotations in mammals, with terrestrial running species having canals that are closer to right angles (90var^38^; e.g., semicircular canal modifications of semi-aquatic otters and minks^26^). This finding contradicts the pattern observed between cheetahs and other felids (i.e., greater angle between ASC and PSC in cheetahs, Supplementary Table 6), but it is possible that the greater vertical development of ASC and PSC in cheetahs has a more important influence than their orthogonality on increasing the afferent vestibular sensitivity to roll and pitch head movements (as radius of curvature R, a measure of canal elongation, is still the most common index used to infer an animal’s agility^21,37^). Moreover, the out-of-plane curvature of ASC and PSC could allow the cheetah to sense accelerations of the head in other directions than the main roll and pitch movements^19,33^. Further physiological studies on the possible effect of semicircular canal curvature on the canal afferent sensitivity would enhance interpretations of their interrelationships.

Postcranial remains of *Acinonyx pardinensis* suggest that fossil cheetahs already were adapted for fast running since at least the beginning of the Pleistocene^39–42^. Nevertheless, the small vestibular system of the fossil cheetah species, and, in particular, the presence of a shorter common crus and less extended ASC and PSC compared to the extant cheetah, indicate a relatively lower afferent sensitivity to angular and linear head movements. In addition to those physiological and functional aspects, the shape patterns of the vestibular system of *A. pardinensis* closely resemble that of the early fossil felid *Proailurus*, reflecting the retention of a morphologically primitive, ancestral condition, and emphasizing that postcranial skeleton modifications for higher speed locomotion preceded adaptations in the inner ear sensory system within the cheetah lineage. The extreme enlargement of the vestibular system and elongation of the ASC and PSC exhibited by modern cheetahs thus appears to be a unique and extremely recent key acquisition to fast running within that lineage. It is possibly more recent than the middle Pleistocene (i.e., 126,000 year old last occurrence of *Acinonyx pardinensis* in the fossil record) and could even coincide with the late Pleistocene population bottleneck for *A. jubatus*, as estimated from gene sequences of modern African cheetahs^10^.

## Methods

### Specimens

We scanned 19 specimens of extant felids, including 13 species across each major lineage^27^ (Supplementary Fig. 1, Supplementary Table 1). The extant cheetah *Acinonyx jubatus* is represented by 7 specimens, including 3 subspecies of the 5 recognized^3^ (Supplementary Table 2). Our extant felid sample encompasses terrestrial and scansorial taxa, one cursorial hunter (cheetah, *Acinonyx jubatus*), one piscivorous cat (fishing cat, *Prionailurus viverrinus*) and three arboreal specialists (margay, *Leopardus wiedii*; marbled cat, *Pardofelis marmorata*; clouded leopard, *Neofelis nebulosa*). Most specimens are from the Mammalogy collections and Vertebrate Paleontology teaching collections of the American Museum of Natural History, New York (AMNH) and were digitized at the AMNH Microscopy and Imaging Facility (MIF) using a high-resolution GE Phoenix Vtome x s240 micro-CT scanner. Two fossil taxa were included in this study, the earliest known felid *Proailurus lemanensis* and the ‘giant’ Pleistocene cheetah *Acinonyx pardinensis*, in order to assess morphological changes of the inner ear through felid evolutionary history and particularly the timing of the onset of the peculiar hunting behavior of modern cheetahs. We scanned the holotype specimen of the type species *Proailurus lemanensis*, recovered from the early Miocene locality of Saint-Gérand-le-Puy in central France^45^ (MN2a of the European Neogene mammalian biochron zonation). The *P. lemanensis* material is stored in the paleontological collections of the Muséum d’Histoire Naturelle (Paris, France), and digital data were acquired at the AST-RX platform of the Museum with a GE Phoenix Vtome x L240 micro-CT scanner. The specimen of *A. pardinensis*, from the paleontological collections of the Musée des Confluences (Lyon, France), is one of the best preserved skulls ever recovered for that species^46,47^. It comes from earliest Pleistocene deposits of Saint Vallier, France (geochronologically dated to ca. 2.4 Ma^48^). That skull was digitized at the Biomaterials Science Centre of the University of Basel, Switzerland with a GE Phoenix Nanotom. Details on all sampled specimens (including collection location, ontogenic stage, geographical origin, and sex) and on CT and 3D reconstruction data (voxel size, voltage and amperage of the electron beam, side of the reconstructed bony labyrinth) are summarized in Supplementary Table 2.

MIMICS16.0 (Materialise NV, Belgium) was used for 3D reconstructions of bony labyrinths and calculations of vestibular system and bony labyrinth volumes, as well as for linear and angular measurements of the vestibular system, which can be used for further comparative studies (Supplementary Tables 3 and 6). The bony channels for the vestibular and cochlear aqueducts were removed for volume measurements, as the membranous structures associated are only partly surrounded by bone and therefore cannot be entirely reconstructed^17^.

### Vestibular and bony labyrinth volume analysis

The ratio of vestibular system volume relative to the volume of the entire bony labyrinth was calculated, and those values were plotted against the phylogenetic tree using the packages APE^49^ and Phytools^50^ in R 3.1.1 (Fig. 1a). The phylogeny is a subset from a felid tree^51^, which was based on molecular data^27^ with inclusion of fossils using stratigraphic occurrences (Supplementary Fig. 1, see Supplementary Table 1 for details on divergence dates). To take into account the possible allometric relationship between body mass and bony labyrinth volume, the log-transformed volume of the vestibular system (in mm^3^) alone was regressed against the log-transformed estimated body mass (in g [grams]) of the felid species using a simple least squares regression model in R (Supplementary Table 4). Estimated body mass was calculated according to an equation based on the condylobasal length of the skull for each specimen^52^. Volumetric and body mass data are reported in Supplementary Table 3. Residuals of the regression model are presented in Supplementary Table 4 and plotted against the phylogenetic tree in Fig. 1b using the same R packages as for the ratios of the vestibular system (APE and Phytools).

### 3D shape analysis

3D geometric morphometric analyses of the vestibular system were performed following the protocol of a previous study^26^: 3 fixed landmarks were placed at the bifurcation points of the semicircular canals and 80 semilandmarks were generated from curves placed at the center of the canals and common crus (20 per canal and 20 for the common crus) using ISE-Meshtools 1.3^53^ (http://morphomuseum.com/meshtools; see Supplementary Table 5 for detailed location of landmarks and semilandmarks). Procrustes superimposition with sliding process minimizing Bending Energy was performed to obtain 3D Procrustes coordinates of the vestibular system, using the R package Geomorph^54^. The configuration of Procrustes coordinates obtained using that sliding process was compared to a generalized Procrustes analysis minimizing Procrustes distance (see statistical tests between the two approaches^26,55,56^). The Bending Energy approach led to a more uniform distribution of coordinates along curves, and therefore was chosen for subsequent analyses. To analyse shape variation of the vestibular system, Principal Components Analysis (PCA) of the Procrustes coordinates of individual felid specimens was performed (Fig. 1c). To test for allometry in the shape data, a Procrustes ANOVA between the Procrustes coordinates (mean Procrustes coordinates for the 7 specimens of *Acinonyx jubatus*) and the log-transformed centroid size was undertaken. The phylogenetic signal in the shape data also was calculated, using the K*mult* statistic, in which the K value (between 0 and 1) reflects lesser or greater correspondence between the observed shapes and those expected from the phylogeny under a Brownian model of evolution^57,58^. The K value was evaluated statistically via permutation tests, with data at the tips of the phylogeny randomized relative to the reference tree.

### Data availability statement

All data generated or analysed during this study are included in this published article (and its Supplementary Information files).

## Electronic supplementary material

Supplementary Information

## References

[1] Sharp NCC (1997). Time running speed of a cheetah (*Acinonyx jubatus*). J. Zool..

[2] Wilson AM (2013). Locomotion dynamics of hunting in wild cheetahs. Nature.

[3] Krausman PR, Morales SM (2005). *Acinonyx jubatus*. Mamm. Species.

[4] Hayward NW, Hofmeyr M, O’Brien J, Kerley GHI (2006). Prey preferences of the cheetah (*Acinonyx jubatus*) (Felidae: Carnivora): morphological limitations or the need to capture rapidly consumable prey before kleptoparasites arrive?. J. Zool..

[5] Kitchener, A. C., Van Valkenburgh, B. & Yamaguchi, N. Felid form and function. In: *Biology and Conservation of Wild Felids* (eds Macdonald, D. W. & Loveridge, A. J.) 83–106 (Oxford University Press, New York, 2010).

[6] Hildebrand M (1961). Further studies on locomotion of the cheetah. J. Mammal..

[7] Kitchener, A. *The Natural History of the Wild Cats* (Christopher Helm, A. & Black, C., London, UK, 1991).

[8] Hudson PE (2011). Functional anatomy of the cheetah (*Acinonyx jubatus*) hindlimb. J. Anat..

[9] Hudson PE (2011). Functional anatomy of the cheetah (*Acinonyx jubatus*) forelimb. J. Anat..

[10] Dobrynin P (2015). Genomic legacy of the African cheetah, *Acinonyx pardinensis*. Genome Biol..

[11] Cox PG, Jeffery N (2007). Morphology of the mammalian vestibulo-ocular reflex: the spatial arrangement of the human fetal semicircular canals and extraocular muscles. J. Morphol..

[12] Cox PG, Jeffery N (2008). Geometry of the semicircular canals and extraocular muscles in rodents, lagomorphs, felids and modern humans. J. Anat..

[13] Schmelzle T, Sanchez-Villagra MR, Maier W (2007). Vestibular labyrinth diversity in diprotodontian marsupial mammals. Mammal. Study.

[14] Lebrun R, de Léon MP, Tafforeau P, Zollikofer C (2010). Deep evolutionary roots of strepsirrhine primate labyrinthine morphology. J. Anat..

[15] Luo Z, Ruf I, Schultz JA, Martin T (2010). Fossil evidence on evolution of inner ear cochlea in Jurassic mammals. Proc. R. Soc. B..

[16] Boistel R (2011). Shake rattle and roll: the bony labyrinth and aerial descent in squamates. Integr. Comp. Biol..

[17] Ekdale EG (2013). Comparative anatomy of the bony labyrinth (inner ear) of placental mammals. PLoS ONE.

[18] Mennecart B (2017). Bony labyrinth morphology clarifies the origin and evolution of deer. Sci. Rep..

[19] Spoor F, Wood B, Zonneveld F (1994). Implications of early hominid labyrinthine morphology for evolution of human bipedal locomotion. Nature.

[20] Spoor F, Bajpai S, Hussain ST, Kumar K, Thewissen JGM (2002). Vestibular evidence for the evolution of aquatic behaviour in early cetaceans. Nature.

[21] Spoor F (2007). The primate semicircular canal system and locomotion. Proc. Natl. Acad. Sci. USA.

[22] Lindenlaub T, Burda H, Nevo E (1995). Convergent evolution of the vestibular organ in the subterranean mole-rats *Cryptomys* and *Spalax*, as compared with the aboveground rat. Rattus. J. Morphol..

[23] Alonso PD, Milner AC, Ketcham RA, Cookson MJ, Rowe TB (2004). The avian nature of the brain and inner ear of *Archaeopteryx*. Nature.

[24] Ladevèze S, de Muizon C, Colbert M, Smith T (2010). 3D computational imaging of the petrosal of a new multituberculate mammal from the Late Cretaceous of China and its paleobiologic inferences. C. R. Palevol..

[25] Ni X, Flynn JJ, Wyss AR (2010). The bony labyrinth of the early platyrrhine primate. Chilecebus. J. Hum. Evol..

[26] Grohé C, Tseng ZJ, Lebrun R, Boistel R, Flynn JJ (2016). Bony labyrinth shape variation in extant Carnivora: a case study of Musteloidea. J. Anat..

[27] Johnson WE (2006). The Late Miocene radiation of modern Felidae: a genetic assessment. Science.

[28] Peigné S (1999). *Proailurus*, l’un des plus anciens Felidae (Carnivora) d’Eurasie: systématique et évolution. Bull. Soc. Hist. Nat. Toulouse.

[29] Müller M (1994). Semicircular duct dimensions and sensitivity of the vertebrate vestibular system. J. Theor. Biol..

[30] Gunz P, Ramsier M, Kuhrig M, Hublin J-J, Spoor F (2012). The mammalian bony labyrinth reconsidered, introducing a comprehensive geometric morphometric approach. J. Anat..

[31] Alloing-Séguier L, Sánchez-Villagra MR, Lee MSY, Lebrun R (2013). The bony labyrinth in diprotodontian marsupial mammals: diversity in extant and extinct forms and relationships with size and phylogeny. J. Mammal. Evol..

[32] Billet G, Hautier L, Lebrun R (2015). Morphological diversity of the bony labyrinth (inner ear) in extant xenarthrans and its relation to phylogeny. J. Mammal..

[33] Le Maître A, Schuetz P, Vignaud P, Brunet M (2017). New data about semicircular canal morphology and locomotion in modern hominoids. J. Anat..

[34] Müller M (1999). Size limitations in semicircular duct systems. J. Theor. Biol..

[35] McVean A (1999). Are the semicircular canals of the European mole, *Talpa europaea*, adapted to a subterranean habitat?. Comp. Biochem. Physiol. A. Mol. Integr. Physiol..

[36] Yang A, Hullar TE (2007). Relationship of semicircular canal size to vestibulo-nerve afferent sensitivity in mammals. J. Neurophysiol..

[37] Cox PG, Jeffery N (2010). Semicircular canals and agility: the influence of size and shape measures. J. Anat..

[38] Malinzak MD, Kay RF, Hullar TE (2012). Locomotor head movements and semicircular canal morphology in primates. Proc. Natl. Acad. Sci. USA.

[39] Kurtén B (1968). Pleistocene Mammals of Europe.

[40] Van Valkenburgh B, Grady F, Kurten B (1990). The Plio-Pleistocene cheetah-like cat *Miracinonyx inexpectatus* of North America. J. Vert. Paleontol..

[41] Turner A, Antón M (1997). The Big Cats and Their Fossil Relatives: an Illustrated Guide to Their Evolution and Natural History.

[42] Hemmer H, Kahlke R-D, Vekua AK (2011). The cheetah *Acinonyx pardinensis* (Croizet et Jobert, 1828) s.l. at the hominin site of Dmanisi (Georgia) – A potential prime meat supplier in Early Pleistocene ecosystems. Quat. Sci. Rev..

[43] Jones GM, Spells KE (1963). A theoretical and comparative study of the functional dependence of the semicircular canal upon its physical dimensions. Proc. R. Soc. London B..

[44] Fitzpatrick RC, Butler JE, Day BL (2006). Resolving head rotation for human bipedalism. Curr. Biol..

[45] Filhol, H. Étude des mammifères fossiles de Saint-Gérand-le-Puy (Allier). *Ann. Sci. Géol. Paris* 1–253 (1879).

[46] Viret J (1954). Le loess à bancs durcis de Saint-Vallier (Drôme) et sa faune de mammifères villafranchiens. Nouvelles Arch. Mus. Hist. Nat. Lyon.

[47] Geraads D (2014). How old is the cheetah skull shape? The case of *Acinonyx pardinensis* (Mammalia, Felidae). Geobios.

[48] Nomade S (2014). ^40^Ar/^39^Ar constraints on some French landmark Late Pliocene to Early Pleistocene large mammalian paleofaunas: paleoenvironmental and paleoecological implications. Quat. Geochronol..

[49] Paradis E, Claude J, Strimmer K (2014). APE: analyses for phylogenetics and evolution in R language. Bioinformatics.

[50] Revell L (2012). J. phytools: An R package for phylogenetic comparative biology (and other things). Methods Ecol. Evol..

[51] Piras P (2013). Bite of the cats: relationships between functional integration and mechanical performance as revealed by mandible geometry. Syst. Biol..

[52] Mazak JH, Christiansen P, Kitchener A (2011). Oldest known pantherine skull and evolution of the tiger. PLoS ONE.

[53] Lebrun, R. ISE-MeshTools, a 3D interactive fossil reconstruction freeware. 12^th^ Annual Meeting of EAVP, Torino, Italy; 06/2014 (2014).

[54] Adams DC, Otarola-Castillo E (2013). Geomorph: an R package for the collection and analysis of geometric morphometric shape data. Methods Ecol. Evol..

[55] Perez SI, Bernal V, Gonzalez PN (2006). Differences between sliding semi-landmark methods in geometric morphometrics, with an application to human craniofacial and dental variation. J. Anat..

[56] Tseng ZJ, Flynn JJ (2015). Are cranial biomechanical simulation data linked to known diets in extant taxa? A method for applying diet-biomechanics linkage models to infer feeding capability of extinct species. PLoS ONE.

[57] Adams DC (2014). A generalized K statistic for estimating phylogenetic signal from shape and other high-dimensional multivariate data. Syst. Biol..

[58] Blomberg, S. P., Garland, T. & Ives, A. R. Testing for phylogenetic signal in comparative data: behavioral traits are more labile. *Evolution* **57,** 717–745 (2003). 10.1111/j.0014-3820.2003.tb00285.x 12778543

