# Supplementary material for: Recent inner ear specialization for high-speed hunting in cheetahs
Source: Sci Rep. 2018 Feb 2;8:2301. doi: 10.1038/s41598-018-20198-3 (PMC5797172; doi:10.1038/s41598-018-20198-3)
Supplement: Supplementary file 1 — Supplementary Information [file 41598_2018_20198_MOESM1_ESM.pdf]

Supporting Information for

Recent inner ear specialization for high-speed hunting in cheetahs

Camille Grohé, Beatrice Lee, and John J. Flynn

Supplementary Figure 1. Consensus phylogeny of Felidae. Node numbers correspond to divergence age estimates in Supplementary Table 1. Stratigraphic occurrences are 23.03-20 Ma (Oligo-Miocene to early Miocene) for *Proailurus lemanensis* and 2.58-0.126 Ma (early-middle Pleistocene) for *Acinonyx pardinensis*. Bony labyrinth representations not to scale.

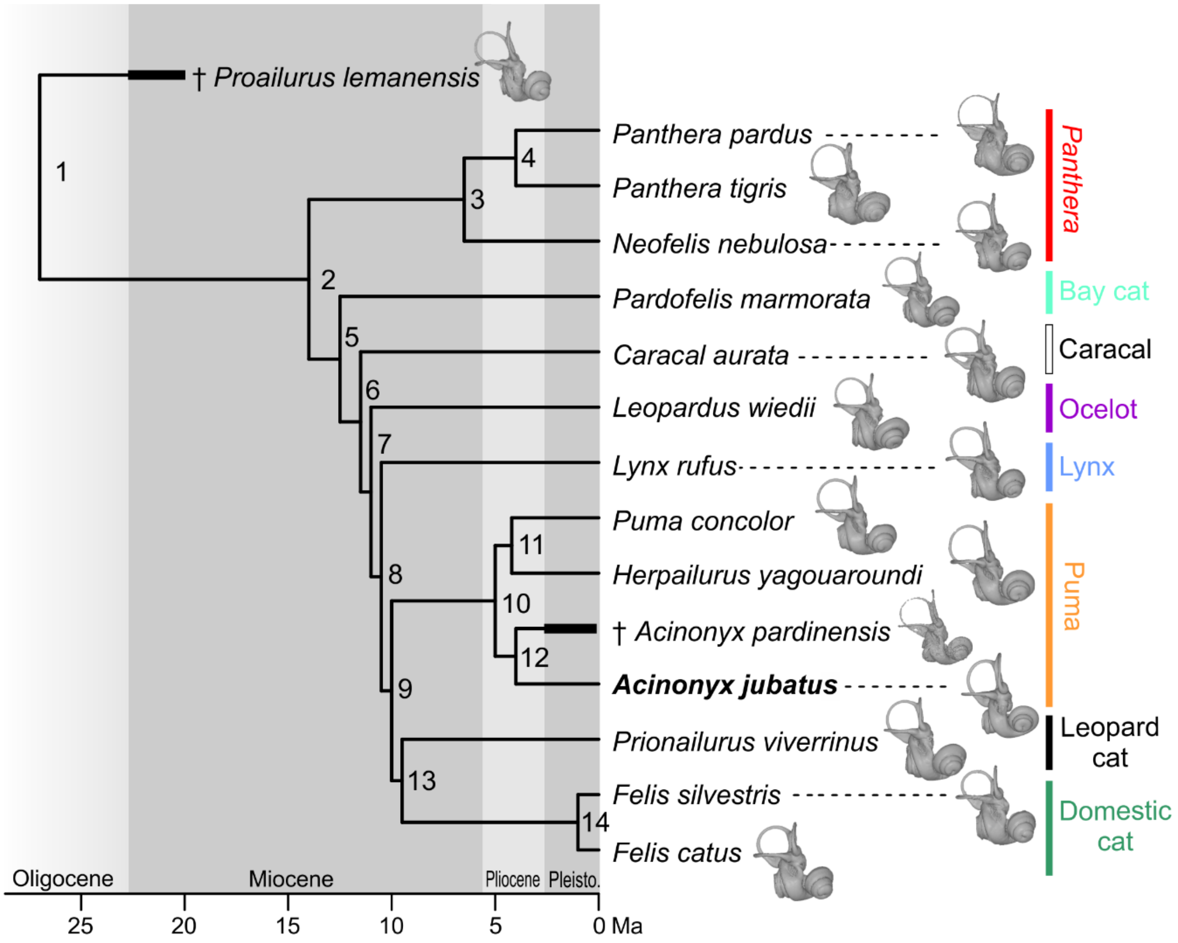

Supplementary Figure 2. Visualization of shape changes in felids for the negative and positive values of PC1. Bony labyrinth in lateral (**a**), dorsal (**b**), posterior (**c**) and anterior (**d**) views (associated with Fig. 1c). Orientation of the bony labyrinth: ant, anterior; dors, dorsal; lat, lateral; post, posterior; ventr, ventral. Arrows highlight shape features discussed in the text.

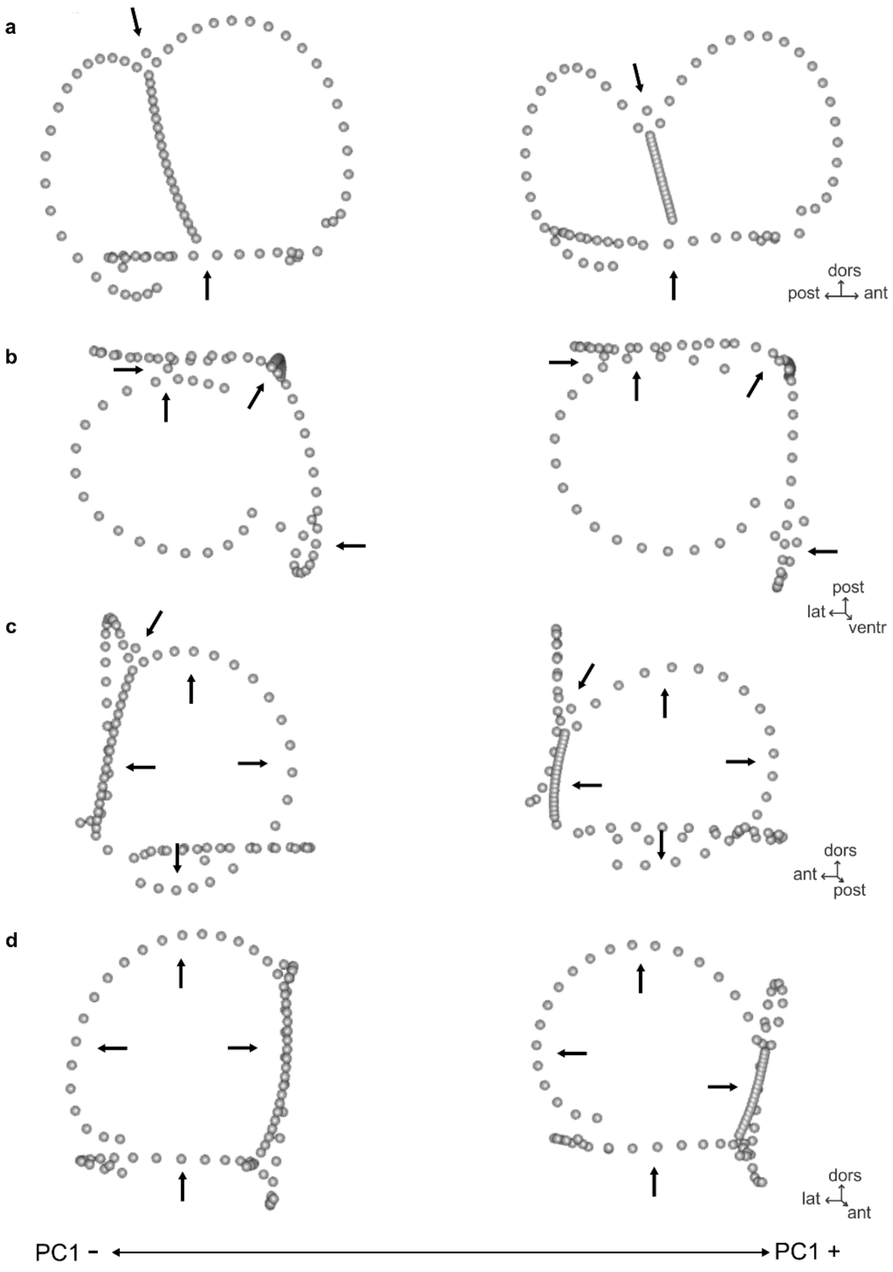

Supplementary Figure 3. Visualization of shape changes in felids for the negative and positive values of PC2. Bony labyrinth in lateroposterior view (associated with Fig. 1c). Orientation of the bony labyrinth: ant, anterior; dors, dorsal; lat, lateral. Arrows highlight shape features discussed in the text.

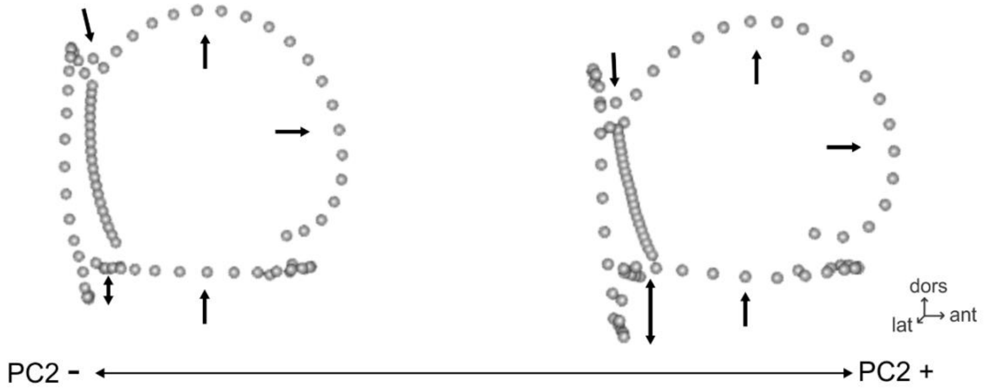

Supplementary Table 1. Divergence age estimates for felids (associated with Supplementary Fig. 1).

Node number (this study)	Node number[1]	Age of nodes (Ma)	References	Descending nodes (NA is used when no OTUs descend from the node)
1	103	**27**	2, 3	*†Proailurus lemanensis*-Felidae
2	135	**14**	3	Felinae
3	170	**6.5**	4	*Panthera-Neofelis*
4	172	**4**	3	*Panthera*
5	136	**12.5**	3, 4	NA
6	137	**11.5**	4	NA
7	138	**11**	4	NA
8	139	**10.5**	4	NA
9	140	**10**	4	NA
10	152	**5**	4	*Puma-Acinonyx-Miracinonyx*
11	156	**4.2**	4	*Herpailurus yagouaroundi*
12	155	**4**	5	*Acinonyx jubatus/†A. pardinensis*
13	141	**9.5**	4	NA
14	147	**1**	4	*Felis catus/F. silvestris*

Reference list

1. Piras, P. *et al.* Bite of the cats: relationships between functional integration and mechanical performance as revealed by mandible geometry. *Syst. Biol.* **62,** 878–900 (2013).

2. Peigné, S. *Proailurus*, l'un des plus anciens Felidae (Carnivora) d'Eurasie: systématique et évolution. *Bull. Soc. Hist. Nat. Toulouse* **135,** 125–134 (1999).

3. Werdelin, L., Yamaguchi, N., Johnson, W. E. & O'Brien, S. J. Phylogeny and evolution of cats. In: *Biology and Conservation of Wild Felids* (eds MacDonald, D. W. & Loveridge, A. J.) 59–82 (Oxford University Press, New York, 2010).

4. Johnson, W. E. *et al.* The Late Miocene radiation of modern Felidae: a genetic assessment. *Science* **311,** 73–77 (2006).

5. Werdelin, L. & Peigné, S. Carnivora. In: *Cenozoic Mammals of Africa* (eds Werdelin, L. & Sanders, W. J.) 603–657 (University of California Press, Berkeley and London, 2010).

Supplementary Table 2. List of the specimens analysed, and associated CT data information. Abbreviations for specimens are: AMNH, American Museum of Natural History, New York; M- and MO-, AMNH Mammalogy collection; FM, AMNH Fossil Mammals collection; F:AM, AMNH Frick Fossil Mammals collection; CCEC, Centre de Conservation et d'Ètude des Collections, Musée des confluences, Lyon, France; MNHN F, Fossils from the Muséum d'Histoire Naturelle of Paris, France; SG-, collection of Saint-Gérand-le-Puy. Subspecies are based on the geographic origin of specimens for *Acinonyx jubatus*[3]. Extinct species are marked by the dagger symbol (*†*). Abbreviations: µm, micrometer; kV, kilovolt; mA, milliampere; L, left; R, right.

Species	Common name	Specimen number	Stage	Voxel size (µm)	Voltage (kV)	Amperage (mA)	Inner ear	Geographical origin	Sex
Acinonyx jubatus	Cheetah	AMNH FM 145070	adult	0.04004	120	180	L	unknown	unknown
Acinonyx jubatus	Cheetah	AMNH FM 145071	adult	0.08042	140	170	R	unknown	unknown
Acinonyx jubatus soemeringii	Northeast cheetah	AMNH M-90255	adult	0.06788	140	170	R	60 miles South of Harrar, Ethiopia	male
Acinonyx jubatus raineyi	Tanzanian cheetah	AMNH M-27897	adult	0.08224	130	170	R	Nyamusi, Nyanza Province, Kenya	male
Acinonyx jubatus raineyi	Tanzanian cheetah	AMNH M-161139	adult	0.06591	130	170	R	25 miles east Ngorongoro Crater, Arusha, Tanzania	male
Acinonyx jubatus jubatus	South African cheetah	AMNH M-119655	adult	0.0646	130	170	R	Tambara District, Mozambique	female

Acinonyx jubatus jubatus	South African cheetah	AMNH M-80618	juvenile	0.06476	130	170	R	Chitau, Bié Province, Angola	female
† Acinonyx pardinensis	‘Giant’ cheetah	CCEC-161825	adult	0.05	180	30	R	Saint Vallier, France	unknown
Caracal aurata	Golden cat	AMNH M-51996	adult	0.05662	130	170	R	Kenge, Democratic Republic of the Congo	male
Felis silvestris lybica	African wildcat	AMNH M-81233	adult	0.04872	130	170	R	Ethiopia	male
Felis catus	Domestic cat	AMNH F:AM 145069	juvenile	0.05077	120	160	R		unknown
Leopardus wiedii	Margay	AMNH M-95085	adult	0.05372	130	170	R	Santarém, Pará state, Brazil	male
Lynx rufus	Bobcat	AMNH M-24225	adult	0.05683	140	170	R	Brownsville, Cameron County, Texas	unknown
Neofelis nebulosa	Clouded leopard	AMNH M-22919	adult	0.09764	170	300	R	Central Park zoo, New York City	unknown
Panthera pardus	Leopard	AMNH M-113745	adult	0.136	160	170	R	Madras, India	male
Panthera tigris	Tiger	AMNH M-45519	adult	0.12025	160	150	R	Futsing, Fujian Province, China	male
Pardofelis marmorata	Marbled cat	AMNH M-102844	adult	0.0484	130	170	R	Sumatra, Indonesia	male
† Proailurus lemanensis		MNHN F-SG3509a	adult	0.05551	125	310	R	Saint-Gerand-Le-Puy, France	unknown
Prionailurus viverrinus	Fishing cat	AMNH M-102691	adult	0.05662	130	170	R	Java, Indonesia	male
Puma concolor	Puma	AMNH MO-6677	adult	0.095	160	180	R	Mascota, Jalisco state, Mexico	male

Herpailurus yagouaroundi	Jaguarundi	AMNH M-24853	adult	0.05419	120	150	R	Escuinapa, Sinaloa state, Mexico	male

Supplementary Table 3. Volumetric measurements of the bony labyrinths and body mass estimation of felid specimens based on the following equation[52]: $\log_{10}BM = (\log_{10}CBL - 1.723)/0.345$, with BM, Body Mass (kg) and CBL, CondyloBasal Length (mm). Vol, Volume (mm^3); BL, Bony Labyrinth; VS, Vestibular System; CS, Cochlear System; % Vol VS= (Vol BL/Vol VS) × 100. BM is in grams. Extinct species are marked by the dagger symbol (†). See Methods for details.

Species	Specimen number	Vol BL	Vol VS	Vol CS	% Vol VS	CBL	BM
Acinonyx jubatus	AMNH FM 145070	109.1	44.83	64.27	41.1	153.6	22037.5
Acinonyx jubatus	AMNH FM 145071	98.78	43.14	55.64	43.7	152	21378.7
Acinonyx jubatus soemerringii	AMNH M-90255	124.13	48.17	75.96	38.8	135.4	15289.8
Acinonyx jubatus raineyi	AMNH M-27897	117.17	44.82	72.35	38.2	192.7	42524.8
Acinonyx jubatus raineyi	AMNH M-161139	120.07	47.06	73.01	39.2	166.93	28049.5
Acinonyx jubatus jubatus	AMNH M-119655	126.52	48.06	78.46	38	168.6	28870.7
Acinonyx jubatus jubatus	AMNH M-80618	127.64	51.35	76.29	40.2	103.1	6939.52
Caracal aurata	AMNH M-51996	96.68	26.78	69.9	27.7	99	6169.46
Felis catus	AMNH F:AM 145069	57.15	19.04	38.11	33.3	61	1515.89
Felis silvestris	AMNH M-81233	71.75	21.68	50.07	30.2	88	4385.09
Herpailurus yagouaroundi	AMNH M-24853	62.53	22.36	40.17	35.8	99.69	6294.92
Leopardus wiedii	AMNH M-95085	73.79	19.56	54.23	26.5	89	4531.09
Lynx rufus	AMNH M-24225	99.75	34.88	64.87	35	120	10774.8
Neofelis nebulosa	AMNH M-22919	105.81	34.97	70.84	33	157.8	23829.9
Panthera pardus	AMNH M-113745	161.22	51.49	109.73	31.9	161.36	25421.8
Panthera tigris	AMNH M-45519	241.99	79.14	162.85	32.7	272	115483
Pardofelis marmorata	AMNH M-102844	66.74	21.23	45.51	31.8	95	5474.31
Prionailurus viverrinus	AMNH M-102691	94.68	27.15	67.53	28.7	117.6	10162
Puma concolor	AMNH MO-6677	149.86	47.76	102.1	31.9	167.7	28426.2
† Acinonyx pardinensis	CCEC-161825	124.41	43.18	81.23	34.7	165	27119.8
† Proailurus lemanensis	MNHN F-SG3509a	62.04	20.16	41.88	32.5	143	17912.2

Supplementary Table 4. Log-transformed Vol VS (Volume of the Vestibular System in mm^3) and BM (Body mass in grams) used for the least squares regression model (see Methods) and associated residuals. The model has the following equation: $\log_{10}$Vol VS= 0.36192 × $\log_{10}$BM + 0.01989. R^2= 0.772; Standard error= 0.05455; p-value= 1.627×10^{-5}. Extinct species are marked by the dagger symbol (*†*).

	**$\log_{10}$BM**	**$\log_{10}$ Vol VS**	**Residuals**
Acinonyx jubatus	4.3194	1.6694	0.0862
Caracal aurata	3.7902	1.4278	0.0362
Felis catus	3.1807	1.2797	0.1086
Felis silvestris	3.642	1.3361	-0.0019
Herpailurus yagouaroundi	3.799	1.3495	-0.0453
Leopardus wiedii	3.6562	1.2914	-0.0518
Lynx rufus	4.0324	1.5426	0.0633
Neofelis nebulosa	4.3771	1.5437	-0.0603
Panthera pardus	4.4052	1.7117	0.0975
Panthera tigris	5.0625	1.8984	0.0463
Pardofelis marmorata	3.7383	1.3269	-0.0459
Prionailurus viverrinus	4.007	1.4338	-0.0363
Puma concolor	4.4537	1.6791	0.0473
† Acinonyx pardinensis	4.4333	1.6353	0.0109
† Proailurus lemanensis	4.2531	1.3045	-0.2547

Supplementary Table 5. Definition of the 3D landmarks and semilandmarks in the geometric morphometric analysis. SC, Semicircular Canal.

No	Type	Definition
1	Landmark	Bifurcation point of the lateral and anterior SC
2	Landmark	Bifurcation point of the lateral and posterior SC
3	Landmark	Bifurcation point of the anterior and posterior SC
4 to 23	Semilandmarks	Curve placed at the center of the anterior SC
24 to 43	Semilandmarks	Curve placed at the center of the lateral SC
44 to 63	Semilandmarks	Curve placed at the center of the posterior SC
64 to 83	Semilandmarks	Curve placed at the center of the common crus

Supplementary Table 6. Linear and angular measurements of the semicircular canals of felid specimens. L, length; W, Width; ASC, Anterior Semicircular Canal; LSC, Lateral Semicircular Canal; PSC, Posterior Semicircular Canal. Extinct species are marked by the dagger symbol (*†*). See Methods for details.

Species	Specimen number	ASC L	ASC W	LSC L	LSC W	PSC L	PSC W	Angle ASC/LSC	Angle LSC/PSC	Angle ASC/PSC
Acinonyx jubatus	AMNH FM 145070	4.97	5.28	4.39	4.14	5.04	4.5	88.63	96.56	113.41
Acinonyx jubatus	AMNH FM 145071	5.15	5.35	4.28	4.18	5.54	4.64	88.62	93.78	109.48
Acinonyx jubatus soemerringii	AMNH M-90255	5.03	5.44	4.41	4.3	5.07	4.44	90.67	96.4	111
Acinonyx jubatus raineyi	AMNH M-27897	5.23	5.36	4.56	4.41	5.21	4.53	88.93	92.69	111.37
Acinonyx jubatus raineyi	AMNH M-161139	5.53	5.53	4.61	4.24	5.37	4.67	90.76	92.61	112.17
Acinonyx jubatus jubatus	AMNH M-119655	5.25	5.34	4.64	4.48	5.22	4.86	88.97	91.04	109.15
Acinonyx jubatus jubatus	AMNH M-80618	5.29	5.31	4.29	4.63	5.46	4.76	91.91	90.41	113.67
Caracal aurata	AMNH M-51996	3.89	4.78	4.5	3.99	4.27	4.24	90.52	87.14	88.81
Felis catus	AMNH F:AM 145069	2.86	3.77	3.47	3.54	3.1	3.37	90.44	95.91	89.55
Felis silvestris	AMNH M-81233	3.15	3.93	3.58	3.64	3.17	3.39	85.51	93.1	88.51
Herpailurus yagouaroundi	AMNH M-24853	3.7	4.14	3.21	3.99	4.15	4.16	89.19	91.32	93.48
Leopardus wiedii	AMNH M-95085	3.62	4.16	3.79	3.49	3.41	3.91	86.24	95.19	90.65
Lynx rufus	AMNH M-24225	4.04	4.88	4.5	3.96	4.43	4.55	87	87.73	89.8
Neofelis nebulosa	AMNH M-22919	4.91	5.56	4.57	4.28	4.92	4.94	86.11	95.7	85.48
Panthera pardus	AMNH M-113745	5.45	6.14	5.21	5.3	5.01	5.68	89.42	84.76	89.27
Panthera tigris	AMNH M-45519	5.39	6.95	6.11	5.95	6.26	6.35	90.15	87.06	96.03
Pardofelis marmorata	AMNH M-102844	3.57	4.3	3.86	3.76	3.78	4.03	89.46	90.28	96.66
Prionailurus viverrinus	AMNH M-102691	3.98	4.26	3.83	3.66	3.93	4	90.67	93.48	89.96
Puma concolor	AMNH MO-6677	4.93	5.96	5	4.69	5.42	5.46	90.21	93.17	92.73
† Acinonyx pardinensis	CCEC-161825	5.56	5.63	5.59	5.22	5.22	5.59	84.76	91.31	92.42
† Proailurus lemanensis	MNHN F-SG3509a	4.42	4.54	3.98	3.52	3.47	4.31	82.755	92.49	91.83
